# Improving the iterative Linear Interaction Energy approach using automated recognition of configurational transitions

**DOI:** 10.1007/s00894-015-2883-y

**Published:** 2016-01-12

**Authors:** C. Ruben Vosmeer, Derk P. Kooi, Luigi Capoferri, Margreet M. Terpstra, Nico P. E. Vermeulen, Daan. P. Geerke

**Affiliations:** AIMMS Division of Molecular Toxicology, Department of Chemistry and Pharmaceutical Sciences, Faculty of Sciences, VU University Amsterdam, De Boelelaan 1083, 1081 HV Amsterdam, The Netherlands

**Keywords:** Molecular Dynamics simulations, Binding free energy prediction, Iterative Linear Interaction Energy approach, Cytochrome P450 2D6

## Abstract

Recently an iterative method was proposed to enhance the accuracy and efficiency of ligand-protein binding affinity prediction through linear interaction energy (LIE) theory. For ligand binding to flexible Cytochrome P450s (CYPs), this method was shown to decrease the root-mean-square error and standard deviation of error prediction by combining interaction energies of simulations starting from different conformations. Thereby, different parts of protein-ligand conformational space are sampled in parallel simulations. The iterative LIE framework relies on the assumption that separate simulations explore different local parts of phase space, and do not show transitions to other parts of configurational space that are already covered in parallel simulations. In this work, a method is proposed to (automatically) detect such transitions during the simulations that are performed to construct LIE models and to predict binding affinities. Using noise-canceling techniques and splines to fit time series of the raw data for the interaction energies, transitions during simulation between different parts of phase space are identified. Boolean selection criteria are then applied to determine which parts of the interaction energy trajectories are to be used as input for the LIE calculations. Here we show that this filtering approach benefits the predictive quality of our previous CYP 2D6-aryloxypropanolamine LIE model. In addition, an analysis is performed of the gain in computational efficiency that can be obtained from monitoring simulations using the proposed filtering method and by prematurely terminating simulations accordingly.

**Electronic supplementary material:**

The online version of this article (doi:10.1007/s00894-015-2883-y) contains supplementary material, which is available to authorized users.

## Introduction

Recently, we explored an iterative linear interaction energy (LIE) method to efficiently predict binding affinities of novel compounds to highly flexible proteins [1, 2]. The framework makes use of the approach of Stjernschantz and Oostenbrink [3] to sample different (relevant) parts of conformational space in multiple (short and parallel) molecular dynamics (MD) simulations. Thereby, the accuracy in computing free energies of binding (Δ*G*_*bind*_) was increased, while simultaneously reducing the computational efforts needed to compute Δ*G*_*bind*_ values. In addition, the method was developed such that intermediate steps can be performed in an automated fashion, making it suited for industrial, (semi-)high-throughput use. The approach relies on the LIE method [4], which has been chosen for its merits to be fast thanks to a scoring component and to be able to include protein flexibility through the underlying MD simulations. The latter is crucial when dealing with flexible and promiscuous proteins such as Cytochrome P450 (CYPs), which can oxidize a broad range of (apolar) compounds [5]. In addition, many CYPs are able to bind ligands in different binding poses as demonstrated by the possibility of several CYPs to oxidize substrates at different atomic sites. In order to account for multiple possible ligand-binding modes (and because *a priori* information on dominant binding modes is often lacking), the iterative LIE approach was used to incorporate results from multiple parallel MD simulations that start from different binding poses [1–3] and protein conformations [1, 2].

The central LIE equation can be formulated as follows [4]:

1 $$\begin{array}{@{}rcl@{}} \Delta G_{calc}&=&\alpha \left( \left\langle V^{vdW}_{lig-surr} \right\rangle_{bound} - \left\langle V^{vdW}_{lig-surr} \right\rangle_{free}\right)\\ &&+\beta \left( \left\langle V^{el}_{lig-surr} \right\rangle_{bound} - \left\langle V^{el}_{lig-surr} \right\rangle_{free}\right)\text{,} \end{array} $$

where *α* and *β* are van der Waals and electrostatic scaling parameters, respectively. Both *α* and *β* are treated as empirical parameters here and have previously been reported to adopt a range of values for human CYPs [1–3, 6, 7]. Note that as in other recent LIE models from our group [1, 2, 7], we did not include an additional offset parameter *γ* for fitting here. $\left \langle V^{vdW}_{lig-surr} \right \rangle $ in Eq. 1 is the MD-averaged van der Waals interaction energy between the ligand and its surroundings (either when bound to the protein (*bound*) or when freely present in the solvent (*free*)). $\left \langle V^{el}_{lig-surr} \right \rangle $ in Eq. 1 is the MD-averaged electrostatic interaction energy between the ligand and its surroundings.

Equation 1 relies on the assumption that average interaction energies are calculated based on sufficient conformational sampling of the complex, i.e., of the relevant ligand-binding poses and protein conformations. However, it is computationally demanding to achieve sufficient sampling for a ligand that is bound to a flexible protein and/or for which multiple binding poses are available.

Using iterative LIE, a set of protein-ligand conformations can be used as starting points for parallel MD simulations. Results from the various simulations of a given ligand-protein complex are then combined by assigning weights *W*_*i*_ to the results from the individual simulations *i* [8]:

2 $$ W_{i}=\frac{e^{\frac{-\Delta G_{calc,i}}{k_{B}T}}}{{\sum\limits_{i}^{N}}e^{\frac{-\Delta G_{calc,i}}{k_{B}T}}}\text{.}  $$

*W*_*i*_ depends on the free energy of binding calculated from the corresponding simulation (Δ*G*_*calc*,*i*_), the temperature *T* and the Boltzmann constant *k*_*B*_. *N* is the total number of simulations of the complex. Introducing the *W*_*i*_’s into the LIE formula results in the following equation [3]:

3 $$\begin{array}{@{}rcl@{}} \Delta G_{calc}\!\!&=&\!\!{\alpha\!\sum\limits_{i}^{N}}\! W_{i}\! \left( \!\left\langle\! V^{vdW}_{lig-surr} \!\right\rangle_{bound,i} \!-\! \left\langle\! V^{vdW}_{lig-surr} \!\right\rangle_{\!\!free}\!\right)\\ &&\!\!+ {\beta\!\sum\limits_{i}^{N}} \!W_{i} \!\left( \!\left\langle \!V^{el}_{lig-surr}\! \right\rangle_{bound,i} \!-\! \left\langle\! V^{el}_{lig-surr} \!\right\rangle_{\!\!free}\!\right)\!. \end{array} $$

Every simulation *i* will cover a local part of conformational space, and the subsequent combination of results from different simulations *i* using Eq. 3 is only valid when there is no overlap in configurational space between the different simulations [8]. Therefore, care should be taken even when combining results from different protein-ligand simulations, because major conformational changes may occur during a simulation. Such transitions should be dealt with in an appropriate way, such that only MD energy trajectories corresponding to separate parts of configurational space will be summed over in Eq. 3. Although the probability of major conformational changes in short simulations is limited, when occurring they can result in inaccurate estimates of $\left \langle V_{lig-surr} \right \rangle $: in that case, average nonbonded interaction energies may significantly deviate from the averages for the separate local parts of conformational space visited before and after the configurational transition.

In the current work, an (automated) analysis tool was designed to detect configurational changes during individual simulations. Based on a set of preoptimized presets, the tool filters MD trajectories in order to include only average interaction energies obtained for local parts of conformational space. The design of this method is presented below, followed by an assessment of its effect on the efficiency and prediction accuracy of the iterative LIE model for aryloxypropanolamine binding to the flexible CYP 2D6 enzyme, as introduced in reference [2].

## Methods

### Simulation settings

Experimental Δ*G*_*bind*_ values for the training of LIE parameters *α* and *β* were derived from inhibition data reported by Vaz et al. [9]. The aryloxypropanolamine ligand training set from our previous work [2] was extended with ligands 1, 2, 4, 9, 11-18, 21, 23-25, 27, and 29-36 from Vaz [9]. The ligands were docked into the two protein conformations of Hritz et al. [10] used in the LIE model presented in reference [2]. Docking of the ligands into the protein structures was performed using PLANTS in combination with the ChemPLP scoring function [11, 12]. Using heavy-atom coordinate based principal component analysis (PCA) and *k*-means clustering [13], up to eight different ligand poses were selected per protein conformation to start MD simulations using GROMACS 4.5.7 [14] in order to calculate $\left \langle V_{lig-surr} \right \rangle _{i}$ values in Eq. 3. The protein was described using the Amber99SB force field, heme parameters were taken from reference [15], and ligand parameters were generated using ACPYPE and the General Amber Force Field [16–18]. The system was solvated in a dodecahedrical box of approximately 20,000 TIP3P water molecules [19] containing six sodium counterions. Hydrogens were converted to heavy hydrogens (with a mass of 4.032 amu) and bonds were constrained using the LINCS algorithm [20], allowing a timestep of 4 fs. As previously [2], a Berendsen thermostat [21] with a coupling time of 0.1 ps was employed to maintain the temperature of the system close to its reference value, using separate temperature baths for the solvent and solute degrees of freedom, and a Berendsen barostat [21] was used to maintain the pressure close to its reference value during *NpT* simulations with a coupling time of 0.5 ps and an isothermal compressibility of 4.5∗10^−5^bar ^−1^. Van der Waals and short-range electrostatic interactions were explicitly evaluated every time step for pairs of atoms that were within a 0.9 nm cut-off by using a grid-based neighbor list that was updated every two time steps. Long-range electrostatic interactions were included by using the smooth particle mesh Ewald method [22] with a maximum fast Fourier transform grid spacing of 0.125 nm for the reciprocal space sum. After steepest-descent energy minimization and thermal equilibration, 0.5 ns equilibration was performed, followed by 1.0 ns production. Note that interaction energies were stored every 2 ps.

Interactions of the unbound ligand with the solvent were evaluated from a single MD simulation per ligand using identical settings. Average interaction energies $\left \langle V_{lig-surr} \right \rangle _{free}$ were calculated by averaging over the complete production run. For this purpose, the unbound ligands were solvated in dodecahedral boxes containing approximately 650 TIP3P water molecules, and no counter-ions were introduced.

### Calculating average interaction energies for local parts of conformational space

The energy trajectories obtained from MD were used to detect protein-ligand configurational changes during simulation. The first step aims at finding transitions between conformations by identifying large and rapid changes in the protein–ligand interaction energies $V^{el}_{lig-surr, bound}$ and $V^{vdW}_{lig-surr, bound}$ during simulation. These changes are typically associated with a change of orientation of the ligand, a change of shape of the active site, or a combination of both. Because it is not always trivial to monitor the relevant orientational changes during simulation, we follow changes in interaction energies over time instead.

**Fig. 1:**
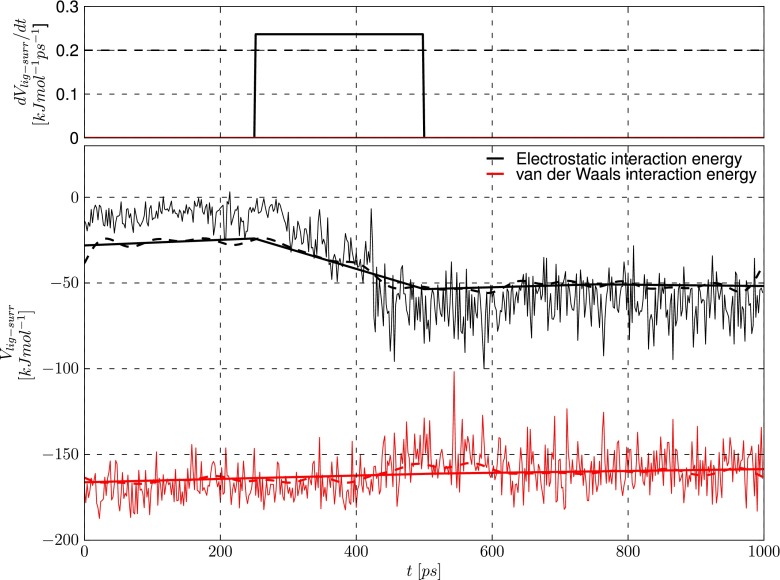
The sequence of filtering steps applied to the raw data for the electrostatic (*black*) and van der Waals (*red*) protein–ligand interaction energies (*thin continuous lines in the lower panel*). The data are first filtered using a Fourier transform (*dashed lines, lower panel*), then splines are fitted to the filtered data (*thick lines, lower panel*). The gradients of the fitted splines are then calculated (*straight lines, upper panel*). A change in conformation is considered to have occurred when the absolute value of the gradient exceeds a predefined cut-off (set to 0.2 kJ mol ^−1^ ps ^−1^, *dashed line, upper panel*)

The raw data for the time series of $V^{el}_{lig-surr, bound}$ and $V^{vdW}_{lig-surr, bound}$ are first processed through a low-pass filter using Fourier transformation to remove high-frequency fluctuations related to thermal fluctuations of the system. Under the assumption that interaction energies fluctuate around constant values between conformational changes, splines are fitted to the Fourier-filtered data. After this step, gradients of the splines are calculated. If the gradient exceeds a preset cut-off, a change in configuration is recorded. Figure 1 illustrates these subsequent steps for an example trajectory.

Once transitions are identified in this way, the average protein–ligand interaction energies that enter (3) are calculated from the raw energy data within the time span that is selected as follows:

1. From the time windows between configurational transitions, the first window with a minimal length *L* is selected. *L* has to be optimized for a given model with respect to the typical frequency of transitions in the individual simulations of the systems.
2. From the selected window, the first time span that is equal to *L* is used.
3. If under (1) no window is found of length *L* or longer, the longest available window is selected. A warning is displayed.

Note that step (2) is introduced in order to investigate how much the computational efficiency of iterative LIE affinity predictions can be further improved. Applying the protocol to typical simulation data as displayed in Fig. 1, two windows are detected ranging from 0−230 ps and 500−1000 ps. With *L* set to 200 ps, interaction energies would be averaged over the first 200 ps of the simulation. With *L*> 230 ps, interaction energy averages would be taken over the according time span that starts at 500 ps.

### Filtering settings

All steps towards detection of configurational transitions from interaction-energy time series were performed using standard Python libraries *numpy* and *scipy*. *scipy.fft* is used to convert the energy data into Fourier space. In Fourier space, only the first 15 elements of the Fourier array are converted back to real space (the complex elements are discarded).

*scipy.interpolate.UnivariateSpline* is used to fit splines, with *k* (degree of smoothing) set to 1, and the positive smoothing factor *s*=999. To obtain the gradient, *numpy.gradient* is used. If the absolute value of the gradient exceeds a cut-off value of 0.2 kJ mol ^−1^ ps ^−1^, a transition is registered (Fig. 1, upper panel).

## Results and discussion

Starting from the two CYP 2D6 structures of Hritz et al. [10] used previously [2], MD simulations and LIE calculations were set up and performed according to the settings described in the Methods section. This setup is optimized to include a larger number of ligands and initial starting poses of the ligand (up to eight per protein conformation in the current work), when compared to the setup used in reference [2]. Hence, an accordingly larger number of simulations is introduced in Eq. 3, which enables optimal exploration of the potential of the filtering methods presented here.

To study the efficiency and accuracy of the proposed filtering method, the minimal length *L* of the time window used to calculate average interaction energies was varied, while values for the gradient cut-off and noise-filtering frequency were maintained identical in all models. The filtered (thermal) noise level and gradient cut-off were optimized here based on visual inspection of the results of the fitting for a small set of selected energy trajectories. In further studies, a way to optimize values for the filtering settings through training, and the subsequent effect on the accuracy and efficiency of the resulting LIE models, could be investigated.

LIE models calibrated using average interaction energies that were obtained by subsequently following steps (1), (2), and (3) as described in the Methods section (with *L*=200, 400, or 600 ps) are presented as ‘filtered’ models in Tables 1 to 3. Root-mean-square errors (RMSEs) and standard deviation of prediction errors (SDEPs) are shown in Table 1, and *α* and *β* values in Table 2. SDEP values were calculated from a Leave-One-Out cross-validation test. The last three columns in Table S1 of the Supplementary Material show that for most compounds *α*, *β* and RMSE of the filtered model with *L* set to 200 ps do not change substantially when leaving out single compounds from the training set.

**Table 1:** Root-mean-square error (RMSE) and standard deviation in error prediction (SDEP) values for LIE models with $\left \langle V^{el}_{lig-surr} \right \rangle _{bound,i}$’s and $\left \langle V^{vdW}_{lig-surr} \right \rangle _{bound,i}$’s in Eq. 3 averaged over various time spans of simulations *i*

*L*	200 ps		400 ps		600 ps		1000 ps	
	RMSE (kJ mol ^−1^)	SDEP (kJ mol ^−1^)	RMSE (kJ mol ^−1^)	SDEP (kJ mol ^−1^)	RMSE (kJ mol ^−1^)	SDEP (kJ mol ^−1^)	RMSE (kJ mol ^−1^)	SDEP (kJ mol ^−1^)
Unfiltered^a^	6.35	8.69	6.05	8.56	6.14	8.50	6.03	8.46
Filtered^b^	5.76	8.41	5.63	8.02	5.69	8.05		
Filter+ext^c^	5.89	8.52	5.68	8.08	5.65	8.05		

^a^ Ligand interaction energies in protein simulations averaged over the time span ranging from 0 ps to *L*

^b^ Time spans selected according to the protocol in the Methods section

^c^ Same as ^b^, but step (2) of the protocol is omitted

**Table 2:** *α* and *β* values for LIE models with $\left \langle V^{el}_{lig-surr} \right \rangle _{bound,i}$’s and $\left \langle V^{vdW}_{lig-surr} \right \rangle _{bound,i}$’s in Eq. 3 averaged over various time spans of simulations *i*

*L*	200 ps		400 ps		600 ps		1000 ps	
	*α*	*β*	*α*	*β*	*α*	*β*	*α*	*β*
Unfiltered^a^	0.442	0.078	0.446	0.080	0.447	0.084	0.448	0.090
Filtered^b^	0.441	0.088	0.444	0.088	0.444	0.088		
Dilter+ext^c^	0.442	0.087	0.445	0.091	0.445	0.090		

^a^ Ligand interaction energies in protein simulations averaged over the time span ranging from 0 ps to *L*

^b^ Time spans selected according to the protocol in the Methods section

^c^ Same as ^b^, but step (2) of the protocol is omitted

**Table 3:** Average time per simulation *i* in Eq. 3 (sim.) needed to calibrate the models reported in Tables 1 and 2

*L*	200 ps		400 ps		600 ps		1000 ps	
Average	used^d^	corr.^e^	used^d^	corr.^e^	used^d^	corr.^e^	used^d^	corr.^e^
sim. time	(ps)	(ps)	(ps)	(ps)	(ps)	(ps)	(ps)	(ps)
Unfiltered^a^	200	200	400	400	600	600	1000	1000
Filtered^b^	194	283	342	656	435	851		
Filter+ext^c^	526	614	539	853	540	957		

^a^ Ligand interaction energies in protein simulations averaged over the time span ranging from 0 ps to *L*

^b^ Time spans selected according to the protocol discussed in the Methods section

^c^ Same as ^b^, but step (2) of the protocol is omitted

^d^ Simulation times are counted from 0 ps until the end of the time span defined in ^b^ or ^c^

^e^ Same as ^d^, but for simulations for which the selected time span is shorter than *L*, 1000 ps was used in the calculation of the average time

The properties of the filtered models were compared with LIE models calibrated using interaction energies averaged over the first 200, 400, and 600 ps of each simulation (referred to as ‘unfiltered’ models in Tables 1 to 3). As a reference, a LIE model is also presented using interaction energies averaged over 1000 ps of the individual production simulations (from hereon referred to as the ‘ns’ model, last column in Tables 1 to 3). Note that the RMSD, SDEP, *α* and *β* values for this model are different from the model presented in reference [2], due to differences in the docking and clustering algorithms used, in the set of training compounds used, and in the force field employed during MD simulations.

In addition to the filtered models, LIE models were calibrated in which step (2) of the protocol in the Methods section was omitted and interaction energies were averaged over the full time window selected under step (1) (or (3)) of the protocol. In the typical example displayed in Fig. 1, this would have as a consequence that for *L* set to 400 ps, interaction energy averages would be taken over the time span ranging from 500 ps to 1000 ps (instead of to 900 ps). In Tables 1 to 3, the models that make use of the extended time window are referred to as ‘filter+ext’.

**Fig. 2:**
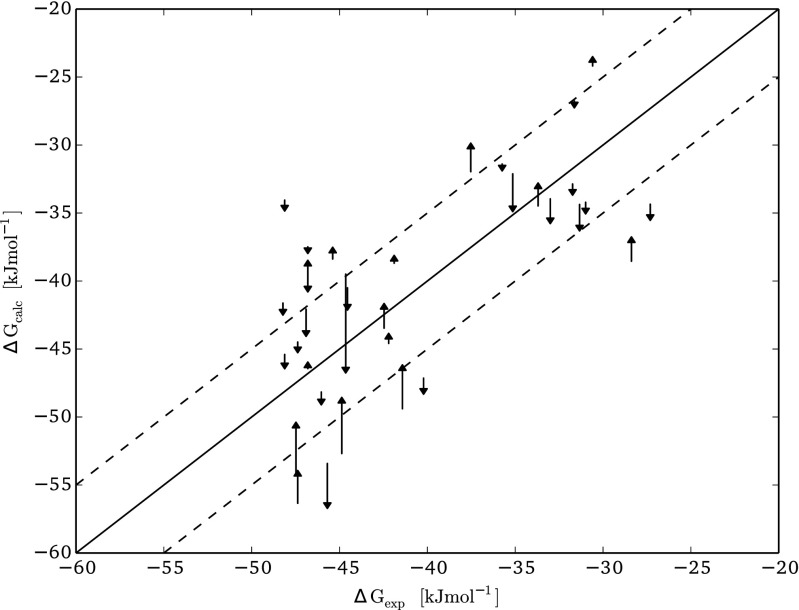
Comparison between the ‘ns’ model and the filtered model with *L* set to 200 ps. The*base of each arrow* is located on the result of the ‘ns’ model, while the *arrow* points at the result of the filtered model

The first line in Table 1 shows that for the unfiltered LIE models, longer sampling times lead to slightly more accurate predictions. With increasing simulation time, RMSE and SDEP values decrease, but the increase in accuracy is limited (with a maximum decrease in RMSE and SDEP values of less than 0.4 kJ mol ^−1^). When considering the filtered and ‘filter+ext’ models (Table 1), the RMSE and SDEP decreased also slightly or adopted similar values with increasing simulation time. Models calibrated using filtered energy trajectories to calculate average interaction energies perform at least as well as the ns model. This is not only demonstrated by the differences in RMSE and SDEP values (Table 1) but also when comparing the correlations between experimental and calculated Δ*G*_*bind*_ values (cf. Fig. 2 and Table S1 of the Supplementary Material, which reports individual values for and errors in the calculated Δ*G*_*bind*_ values). This indicates that, as expected, the noise due to possible conformational changes during simulation is reduced. In general, the filtering has a positive impact on the affinity prediction for individual compounds, as illustrated in Fig. 2 for the filtered model with *L*=200 ps. Although predictions for some ligands become less accurate upon recalibration of the ns model, after filtering the energy trajectories, several ligands for which the prediction by the ns model deviates more than 5 kJ mol ^−1^ from experiment are predicted with increased accuracy in the filtered models.

Table 2 shows that in terms of *α* and *β* value, the filtered models are similar to the unfiltered ones: the filtered models have *α* and *β* values within 1-2 % of the ns model. In addition, the similarity of the three filtered models (Table 2) indicates that their *α* and *β* values are less sensitive to the length of the simulations used than for the unfiltered models. The similarity between the filtered and ‘filter+ext’ models shows as well that once a time window is selected, the length *L* (i.e., length of local sampling) is of limited influence on model calibration. Upon filtering, only windows are used during which interaction energies (thermally) fluctuate around a relatively constant value. Therefore, the average values for the energies are decoupled from the degree of sampling during the individual simulations *i* in Eq. 3. In conclusion, filtering allows to use shorter simulations to calibrate iterative LIE models, without negatively influencing the quality of the model.

In order to evaluate the gain in computational efficiency by using our filtering approach, the simulation times needed to develop the models are summarized in Table 3. For every simulation *i* in Eq. 3, the average simulation time needed to evaluate $\left \langle V^{el}_{lig-surr} \right \rangle _{bound,i}$ and $\left \langle V^{vdW}_{lig-surr} \right \rangle _{bound,i}$ includes the time before accessing the time span (over which interactions are averaged) and the time span itself. In practice, in the case that no window could be selected with a length ≥*L* (step (3) in the protocol in the Methods section), it is only possible to conclude that all time windows are shorter than *L* once the time of simulation *i* reaches 1000 ps. For this reason, Table 3 also reports corrected average simulation times (corr.) that include the full length of the simulation in those cases. The corrected time is representative for the average over individual simulation times needed to train the filtered (or ‘filter+ext’) models. From Table 3, the average simulation time needed to calibrate the filtered model with *L*=200 ps is only 28 % of the time needed for the ns model, corresponding to a gain in efficiency of 72 %. For *L*=400 ps, a gain of 34 % was obtained. Note that for the system studied here, this represents a reduced computational effort of 300 and 100 ns less simulation time in total, respectively. Looking in detail at the number of simulations for which a window of at least length *L* was found according to the protocol in the Methods section, our data show that for more than 90 % of the individual simulations a time span with *L*=200 ps could be found. For 57 % and 37 % of the simulations, a time span of 400 ps or 600 ps was found, respectively. This correlates with the probability of finding a time span of a given length within a simulation of fixed length, under the assumption of random transition frequencies and occurrences. In addition, it demonstrates that for the CYP 2D6-aryloxypropanolamine system, especially for relatively small *L* (200 ps), a significant gain in computational efficiency could be obtained.

## Conclusions

This study shows that through filtering of the interaction energy trajectories used to develop an iterative LIE model, it is possible to improve its predictive quality and efficiency. For the considered system (a set of aryloxypropanolamines binding to Cytochrome P450 (CYP) 2D6) the improvement in accuracy is limited, with a decrease in root-mean-square error and standard deviation of error prediction of less than 0.4 kJ mol ^−1^. A key advantage of using the filtering approach is the substantial decrease in (simulation) time needed to calibrate an iterative LIE model and to predict affinities with the model. Implementing the proposed filtering protocol to assess during simulation whether configurational transitions are occurring (thereby allowing the simulation to be terminated prematurely if sufficient sampling is achieved) would make it possible to significantly reduce the required simulation time.

For the CYP 2D6 protein and aryloxypropanolamine ligand set, it was shown that a model can be calibrated using filtered energy trajectories with a prediction accuracy that is similar to or slightly better than the unfiltered model based on full-nanosecond simulations. Using a 400 ps window, a model with slightly decreased SDEP and RMSE can be proposed that has comparable *α* and *β* parameters, but only carries 66 % of the original costs. Using a 200 ps window, the quality improvement is slightly less, but a reduction of computational cost of 72 % can even be achieved.

An additional gain in computational efficiency could be achieved by applying the filtering protocol already during the equilibration phase of the molecular dynamics (MD) simulation. Currently, a 0.5 ns equilibration run is performed before starting to collect interaction energies from MD. In some cases, an equilibrated conformational state is reached before the end of the equilibration phase, whereas in other cases, parts of the production phase are also needed in addition to reach this conformation. The filtering tool is designed to detect converged parts of interaction energies, which could be indiscriminately applied to the equilibration or the production step of the simulation, thereby possibly further decreasing simulation times needed for predictions by and calibration of iterative LIE models.

## Electronic supplementary material

(PDF 21.1 KB)
