# Supplementary material for: Improving the iterative Linear Interaction Energy approach using automated recognition of configurational transitions
Source: J Mol Model. 2016 Jan 12;22:31. doi: 10.1007/s00894-015-2883-y (PMC4710667; doi:10.1007/s00894-015-2883-y)
Supplement: Supplementary file 1 — (PDF 21.1 KB) [file 894_2015_2883_MOESM1_ESM.pdf]

Table S1: Experimental binding free energies ΔG_{exp} (kJ mol^{-1}) as derived from inhibition data reported by Vaz *et al.* [9] for individual training compounds (numbered here according to the compound indices used by Vaz *et al.*). In addition, calculated values ΔG_{calc} (kJ mol^{-1}) are reported using either $\left\langle V^{el}_{lig-surr} \right\rangle$'s and $\left\langle V^{vdW}_{lig-surr} \right\rangle$'s in Equation (3) averaged over full production runs (ns) or over time spans selected according to the protocol described in the Methods section with $L = 200$ ps, together with their corresponding difference from experiment ($\Delta\Delta G = \Delta G_{exp} - \Delta G_{calc}$). RMSE (kJ mol^{-1}), α and β values are also reported for the filtered LIE models (with L set to 200 ps) in which the corresponding single training compound was left out from model training.

Compound	ΔG_{exp}	ΔG_{calc} (ns)	$\Delta\Delta G$ (ns)	ΔG_{calc} (filt.)	$\Delta\Delta G$ (filt.)	RMSE	α	β
1	-31.7	-32.9	1.1	-33.3	1.6	5.84	0.442	0.089
2	-28.4	-38.5	10.2	-37.2	8.8	5.65	0.443	0.092
3	-31.3	-34.4	3.0	-35.9	4.6	5.79	0.442	0.094
4	-35.7	-31.4	-4.3	-31.5	-4.2	5.79	0.441	0.085
5	-48.2	-41.6	-6.6	-42.1	-6.1	5.74	0.439	0.088
6	-31.7	-26.8	-5.0	-26.9	-4.9	5.77	0.441	0.083
7	-37.5	-32.0	-5.6	-30.3	-7.3	5.70	0.440	0.085
8	-27.3	-34.4	7.0	-35.1	7.8	5.67	0.444	0.086
9	-31.0	-34.2	3.2	-34.7	3.7	5.81	0.442	0.087
10	-30.6	-24.2	-6.4	-23.9	-6.7	5.71	0.441	0.082
11	-33.0	-34.0	1.0	-35.4	2.4	5.83	0.442	0.088
12	-41.4	-49.4	8.0	-46.6	5.1	5.77	0.443	0.086
13	-46.9	-42.1	-4.8	-43.7	-3.2	5.82	0.440	0.088
14	-48.2	-34.1	-14.2	-34.5	-13.8	5.32	0.438	0.088
15	-33.7	-34.5	0.8	-33.2	-0.5	5.84	0.441	0.088
16	-45.7	-53.4	7.7	-56.3	10.6	5.53	0.446	0.090
17	-46.9	-40.1	-6.8	-38.9	-8.0	5.66	0.439	0.085
18	-42.2	-44.6	2.4	-44.3	2.0	5.83	0.442	0.089
21	-46.9	-46.4	-0.5	-46.4	-0.5	5.84	0.441	0.088
22	-42.5	-43.5	1.0	-42.1	-0.4	5.84	0.441	0.087
23	-44.6	-39.5	-5.2	-46.4	1.7	5.83	0.442	0.086
24	-46.9	-40.1	-6.8	-40.4	-6.5	5.73	0.439	0.088
25	-35.1	-32.1	-3.0	-34.5	-0.6	5.84	0.441	0.088
26	-46.9	-37.5	-9.4	-37.6	-9.3	5.61	0.439	0.089
27	-47.5	-54.2	6.7	-50.8	3.3	5.81	0.443	0.087
28	-41.9	-38.7	-3.2	-38.5	-3.4	5.83	0.440	0.094
29	-46.0	-48.2	2.1	-48.7	2.7	5.82	0.442	0.087
30	-47.5	-56.3	8.9	-54.3	6.9	5.70	0.445	0.083
31	-44.9	-52.7	7.8	-49.0	4.1	5.80	0.443	0.089
32	-45.4	-38.4	-7.0	-37.9	-7.5	5.68	0.440	0.084
33	-44.6	-40.5	-4.1	-41.7	-2.9	5.83	0.440	0.090
34	-48.2	-45.4	-2.8	-46.1	-2.2	5.83	0.441	0.087
35	-47.5	-44.5	-3.0	-44.9	-2.6	5.83	0.441	0.088
36	-40.2	-47.1	6.9	-47.9	7.7	5.69	0.444	0.096
